# Bonded Repair Optimization of Cracked Aluminum Alloy Plate by Microwave Cured Carbon-Aramid Fiber/Epoxy Sandwich Composite Patch

**DOI:** 10.3390/ma12101655

**Published:** 2019-05-21

**Authors:** Xiaoyan Liu, Jiacheng Wu, Jiaojiao Xi, Zhiqiang Yu

**Affiliations:** Department of Materials Science, Fudan University, No. 220, Road Handan, Shanghai 200433, China; 15210300041@fudan.edu.cn (X.L.); 17210300052@fudan.edu.cn (J.W.); 16210300028@fudan.edu.cn (J.X.)

**Keywords:** carbon-aramid fiber/epoxy sandwich composite patch, bonded repair, microwave curing, mechanical performance, repair efficiency

## Abstract

Fiber-reinforced epoxy sandwich composites, which were designed as the bonded repair patches to better recover the mechanical performance of a central cracked aluminum alloy plate, were layered by carbon and aramid fiber layers jointly and cured by microwave method in this study. The static tensile and bending properties of both carbon-aramid fiber/epoxy sandwich composite patches and the cracked aluminum alloy plates after bonded repair were systematically investigated. By comparing the mechanical performance with traditional single carbon-fiber-reinforced composite patches, it can be found that the bending performance of carbon-aramid fiber sandwich composite patches was effectively improved after incorporation of flexible aramid fiber layers into the carbon fiber layers, but the tensile strength of sandwich composite patches was weakened to some extent. Especially, the sandwich patches with 3 fiber layers exhibited better tensile and bending performance in comparison to patches of 5 and 7 fiber layers. The optimized 3-layer carbon-aramid fiber sandwich patch repaired plate recovered 86% and 190% of the tensile and bending performance in comparison to the uncracked ones, respectively, showing a considerable repair majorization effect for the cracked aluminum alloy plate.

## 1. Introduction

As one of the most advanced and promising repair methods in recent years, bonded composite repair technology can be widely applied in engineering fields, such as the aerospace industry, the machinery industry, naval architecture, and the construction industry, providing optimal solutions for the quick and qualified fixing of cracked structures [1,2,3,4,5]. With the advantages of low attaching weight, a convenient operating process, good formability, and high repair efficiency, bonded composite repair technology can achieve expected repair goals in practical circumstances well, showing superiority in engineering applications compared with normal repair technologies.

**Figure 1:**
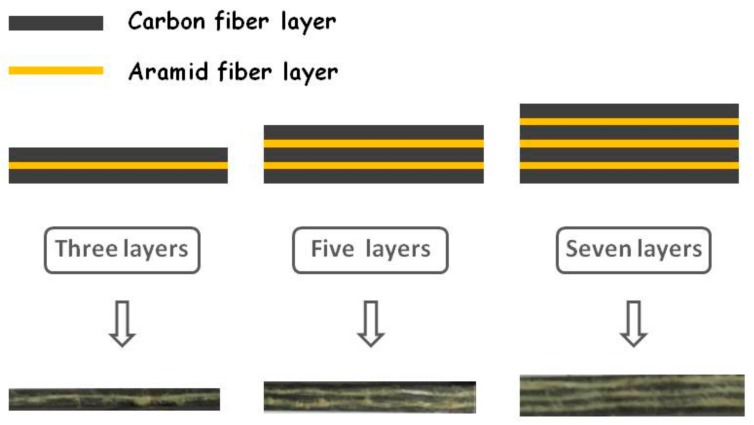
Schematic diagram of the carbon-aramid fiber sandwich composite.

The design and preparation of composite patches, as the vital techniques in bonded composite repair technology, can significantly affect the final repair efficiency of the cracked structures. Composites with different raw material components, various preparation methods, and diverse internal and external designs have been widely investigated and used as repair patches in recent studies [6,7,8,9]. It is noted that composite patches reinforced by fibers show good potential in bonded composite repair technology due to their excellent mechanical behavior, good chemical inactivity, and favorable processability. Various types of fibers, such as carbon fiber, boron fiber, glass fiber, and aramid fiber, were introduced to the matrix to prepare composites with high performance [10,11,12,13,14]. Also, some high performance fibers, such as carbon nanotube fibers [15,16], exhibit excellent mechanical properties and thermal properties, which have the outstanding potential to prepare high performance composites in some specific fields. Among these types of fibers, carbon fiber, which has the advantages of high tensile strength, high tensile modulus, high temperature resistance, good corrosion stability, and low thermal expansion coefficient, is the most widely and effectively used in fiber-reinforced composites. Manalo et al. [10] prepared and applied a pre-impregnated carbon-fiber-reinforced epoxy repair system to the repair of a damaged steel pipe and achieved a good mechanical strengthening effect. Albedah et al. [17] used carbon-fiber-reinforced composite patches to repair a cracked aluminum alloy 7075-T6 plate and found that the fatigue life of the repair structure was effectively increased. Caminero et al. [18,19] also successfully utilized one-sided and two-sided carbon-fiber-reinforced composite patches to repair an impact damaged carbon-fiber-reinforced plastic panel, and digital image correlation techniques were employed to analyze the damage to composite-repaired structures. As with carbon fiber, aramid fiber has also attracted wide attention from researchers recently due to its outstanding mechanical and chemical characterizations. Besides the good strength, high modulus, and stable chemical properties similar to carbon fiber, aramid fiber has the characteristics of commendable flexibility, is comparably lightweight, and smaller in size in comparison with carbon fiber, which means the composite has good deformability as well as excellent bending performance. In the repair process, it is known that the stress distribution around the cracked area is quite complex. Hence, not only the tensile properties but also the bending properties of the composite patch impact the final repair effect of cracks. Under circumstances where the strength of the composite patches is guaranteed, patches with good bending performance can maintain better adhesivity with cracked structures and adapt well to the loading vectors in various planes and directions, improving the suitability of bonded repair structures and enhancing the repair efficiency effectively. Thus, strengthening the bending properties of a composite patch on the basis of guaranteed mechanical strength could be one of the meaningful and applicable methods to further optimize the bonded composite repair technology. Based upon this, it is feasible to join together carbon fiber, which displays excellent mechanical strength, and aramid fiber, which has preferable small size and good flexibility, to prepare a fiber-reinforced composite patch with increased bending capability. In this study, carbon fibers and aramid fibers were designed to be layered alternately to prepare multi-layer composite patches, which take on the shape of a sandwich, displayed in Figure 1. As shown in the cross-section, an aramid fiber layer is located between two carbon fiber layers, and the interlayer aramid fibers provide certain flexibility for the composite patches on shear planes, improving the ability for shear deformation of the composites. On the other hand, the carbon fiber layers offer the main mechanical strength of the composite patches.

As another significant factor during the design and processing of composite patches, the curing method has a significant impact on the final properties of composite patches. Microwave curing, with the advantages of fast heating rate, good radiation uniformity, and favorable energy controllability, is one of the most promising curing methods, with high efficiency and environmentally-friendly characteristics. Nowadays, the microwave curing method has already been applied in the preparation of resin materials due to these valuable features. Boey et al. [20] used the microwave curing method to cure an epoxy–amine system, and finally prepared the uniformly-cured product with high glass-transition temperature successfully. Sung et al. [21] also used microwaves to investigate a curing method that can cure epoxy resin uniformly, where the goals were ultimately achieved as well.

In this paper, the fiber-reinforced laminar composite patches, which were layered with carbon and carbon-aramid fibers and prepared by microwave curing method, were applied to the repair of a central cracked aluminum alloy plate. The static tensile and bending properties were utilized to evaluate the mechanical performance of carbon and carbon-aramid-fiber-reinforced composite patches with different layer numbers. By conducting comparisons repair efficiencies of a cracked aluminum alloy plate between carbon and carbon-aramid fiber jointly-reinforced composite patches, the repair majorization effect of the sandwich composite was further studied and discussed.

## 2. Experimental

### 2.1. Preparation of Fiber-Reinforced Laminar Composites

The hand lay-up molding method was utilized to prepare the fiber-reinforced composites in this study. The carbon fiber fabric (UT70-30, tensile strength 3959 MPa, interlaminar shear strength 49.1 MPa, supplied by Japan Toray Company, Tokyo, Japan) and the aramid fiber fabric (CAS280, tensile strength 2300 MPa, bending strength ≥400 MPa, interlaminar shear strength ≥40 MPa, offered by CARBON Company, Tianjin, China) were tailored in advance to fit the size of the mold, which was 110 mm and 230 mm in width and length, respectively. For easy processing, an appropriate amount of E-51 epoxy resin (colorless viscous liquid, density 1.15–1.2 g/cm^3^, epoxy equivalent 0.48–0.54, provided by Kunshan Lvxun Electronic Co. Ltd., Kunshan, China) was immediately heated by microwave before preparation in order to eliminate the air inside the resin and reduce its viscosity at the same time. After that, the curing agent triethylenetetramine (light yellow oily liquid, density 0.977–0.990 g/cm^3^, Sinopharm Chemical Reagent Co. Ltd. (Shanghai, China)) was introduced into the epoxy matrix, of which the mass ratio is 15% regarding the epoxy resin. After mechanical stirring for 5 min, the mixture was then poured into the mold, and the hand lay-up operations were conducted according to different designs for the fiber types and layer conditions. Subsequently, together with the mold, the processed composite was transferred into the microwave oven to be cured under certain external prestress. The average radiation curing period was 30 s for each cycle, and there were 2 five-second intervals and 3 cycles in total. In particular, to avoid the electric discharge phenomenon during the curing process, the carbon fiber fabrics should be adhered with conducted tapes around the edges. All of the composite plates were finally cut into small square pieces of 40 mm × 40 mm in size for further preparation of the repair patches.

### 2.2. Repair Process of Central Cracked Aluminum Alloy Plate

**Figure 2:**
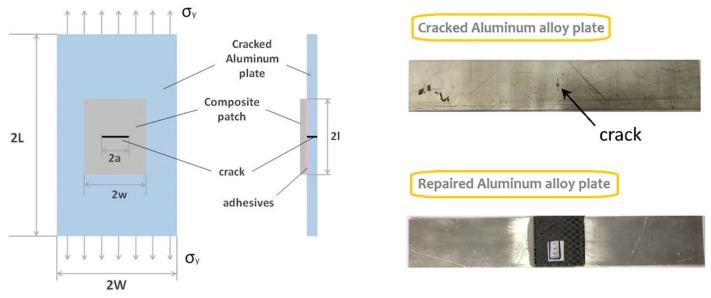
Schematic diagram and samples of bonded composite repair structures.

The schematic diagram of the bonded composite repair technology is shown in Figure 2. As demonstrated, the composite repair patch is bonded onto the cracked plate by the adhesive layer, covering the whole cracked area. In order to achieve better adhesive performance, surface treatments were conducted on the central cracked area of the aluminum alloy plate. The bonding area of the cracked plate was polished with sandpaper and cleaned with water before the repair operations. The adhesive that was used to bond the repair patch and cracked plate together should also be prepared beforehand, according to our previous research [22]. Then, the appropriate amount of the adhesive was smeared uniformly onto the bonding area of the aluminum alloy plate, while the composite repair patch was attached by pressing down slightly. After that, the bonded repair structures were placed into the curing oven and kept for 1 h until the adhesive layer was completely cured.

### 2.3. Characterization

The static mechanical performance, including the tensile and bending performance of the fiber-reinforced composites, as well as bonded repair structures, were conducted on an electronic universal testing machine (INSTRON-6025, INSTRON Company, Boston, MA, America). Tensile properties of fiber-reinforced composites were carried out according to GB/T 3354-1999 with a loading speed of 1 mm/min. The tensile specimen was 230 mm × 15 mm. Bending properties were tested according to the GB/T 1449-2005 at a loading speed of 4 mm/min. The size of the bending specimen was 15 mm × 80 mm. The distance between the two supporting points was 40 mm.

After being sprayed in a gold film, the fracture morphology of the composite was observed by scanning electronic microscope (XL30FEG, Philips, Amsterdam, the Netherlands).

## 3. Results and Discussion

### 3.1. Bending Performance of Carbon-Aramid Fiber/Epoxy Sandwich Composite Patches

**Figure 3:**
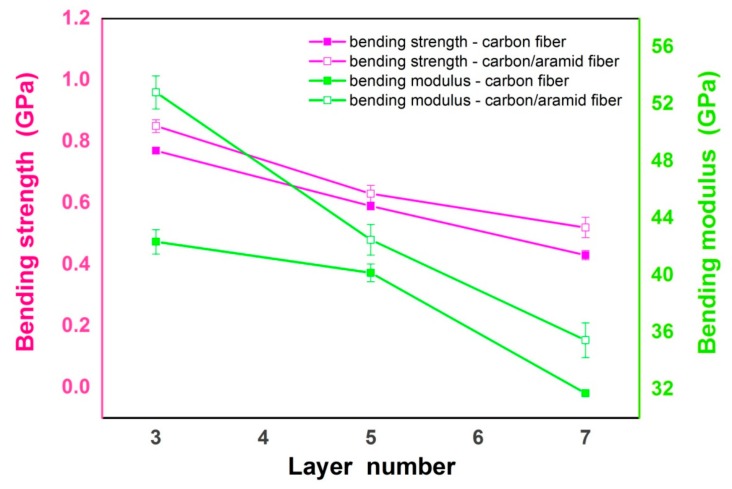
Bending performance of carbon fiber/epoxy composites and carbon-aramid fiber/epoxy sandwich composites with different layer numbers.

In this study, the aramid fiber fabric was designed as the shear-strengthening layer of the laminar composite to improve the shear behaviors of the bonded composite patch. The bending experiments were utilized to characterize the shear behaviors of the bonded composite patch. According to the results of the bending experiment presented in Figure 3, it is easy to find that the design of the carbon-aramid fiber sandwich structure can improve the bending performance of the laminar composite patches. As shown, with the incorporation of aramid fiber layers into the carbon-fiber-reinforced composite, the bending strength, as well as the bending modulus, of the composite patches are increased obviously compared with the traditional composites reinforced by a single carbon fiber fabric. In particular, the bending modulus of the composites with the sandwich structure were improved more effectively, implying the favorable bending properties of the patches and good strengthening effect after the insertion of aramid fiber layers. With low thickness and good flexibility, aramid fiber layers inside a laminar composite are excellent components that can provide the advantageous ability of better bending resistance for the bonded patch under different bending loads. On the other hand, the sandwich structure of the composite can reduce the total thickness of the patch to a certain degree due to the lower density of the aramid fiber than the carbon fiber, contributing to enhancing the bending strength of the composite at the same time. Besides, it can also be observed from Figure 3 that for both the traditional and sandwich composite patches, their bending strength and bending modulus values decline with the increase of fiber layer numbers. In comparison, the composite patches with three fiber fabric layers present the best bending properties. For the laminar composites reinforced by multiple fiber fabric layers, it is noted that the interface conditions among layers inside the composite would be the weak spots of the patch under external shear forces. There is a higher possibility for the formation of defects, such as air bubbles, which would be easily introduced into the composites during the hand lay-up molding process, to exist in the matrix or around the fibers. Thus, with the increase of fiber layers, the possible defects, as well as weak spots inside the composites, increase at the same time, negatively affecting the bending performance of the composites to a large extent. Based on the relationship between bending performance and fiber layer numbers of the laminar mentioned above, the decreasing trends of bending strength and bending modulus, which are observed in Figure 3, can be reasonably explained. Considering that the content of the aramid fiber in the three-layer sandwich patch is smallest compared to the others, which means it contains the smallest interface area within the patch, the negative impact of the incorporation of aramid fibers mentioned above would be most-limited in three-layer sandwich patch in this study, making it the best candidate for improving bending performance. What is more, the carbon fiber content of the three-layer sandwich composite patch is 2/3, higher than 3/5, and 4/7 for the five-layer and seven-layer patches, respectively, which also contributes to the strengthening effect against bending load due to the high strength and modulus of carbon fiber.

### 3.2. Tensile Performance of Carbon-Aramid Fiber/Epoxy Sandwich Composite Patches

**Figure 4:**
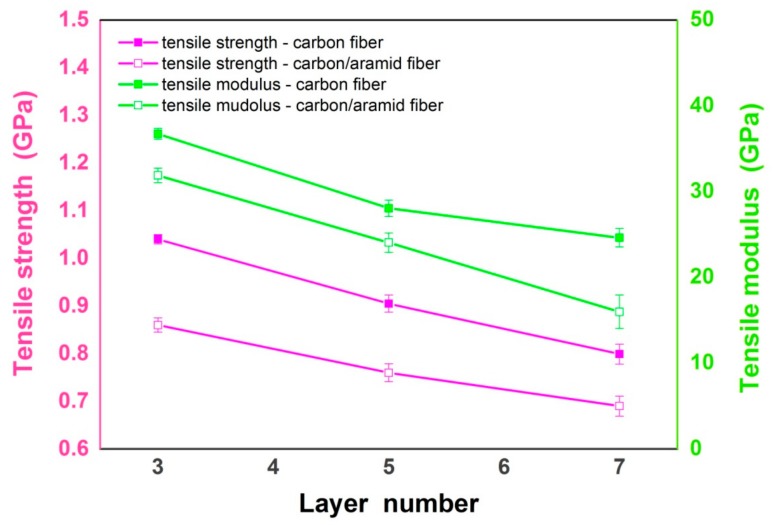
Tensile performance of carbon fiber/epoxy composites and carbon-aramid fiber/epoxy sandwich composites with different layer numbers.

As important factors which impact the mechanical properties of composite patches, not only bending performance but also tensile performance should be investigated to evaluate the overall performance of the composites. The tensile performance variation trend of traditional carbon-fiber-reinforced composites and carbon-aramid-fiber-reinforced sandwich composites are displayed in Figure 4. It can be observed from the chart that unlike the bending performance, the tensile strength and modulus of carbon-aramid-fiber-reinforced sandwich composite patches are lower than those of the carbon-fiber-reinforced ones. This phenomenon implies that the incorporation of flexible aramid fiber layers would impair the tensile properties of the sandwich composites to some extent. According to the mechanical parameters provided by the manufacturers mentioned above, it is noted that for original fiber, the tensile properties of carbon fiber are stronger than those of aramid fiber. Therefore, when the carbon fiber layers inside the composite are partly replaced by the aramid fibers, the overall tensile strength of the composite would ultimately be weakened. On the other hand, that means the positive effect of aramid fiber layers on improving the bending performance of the composite patch could be achieved, however, at the cost of weakening the tensile behaviors of the composite patch simultaneously. Similar to bending performance, from Figure 4, it also indicates that for both traditional and sandwich composite patches, although the total amount of fibers inside the composite patch increases with the addition of fiber layers, their tensile strength, as well as tensile modulus, are decreased adversely. One of the reasonable explanations for this phenomenon is that the weakening effect caused by fiber layer number is closely related to the artificial defects introduced during the process of hand lay-up molding, which is similar to the decreased trend displayed in Figure 3; that is, with the increase of fiber layers, there is a high and increasing possibility for the weak spots to be produced among fiber layers inside the composites. When the composite patches are stretched under tensile loading, the air bubbles inside become the stress concentration spots. The cracks further generate around the defects, leading to the damage of these areas and ultimately the failure of the whole patch. Therefore, with the increase of defects, the tensile strength of the patch is negatively impacted. Additionally, the increasing thickness of the composite, which enlarges the cross sectional area of the composite patch, also contributes to the decrease of tensile strength with the increase of fiber layers inside the patch.

**Figure 5:**
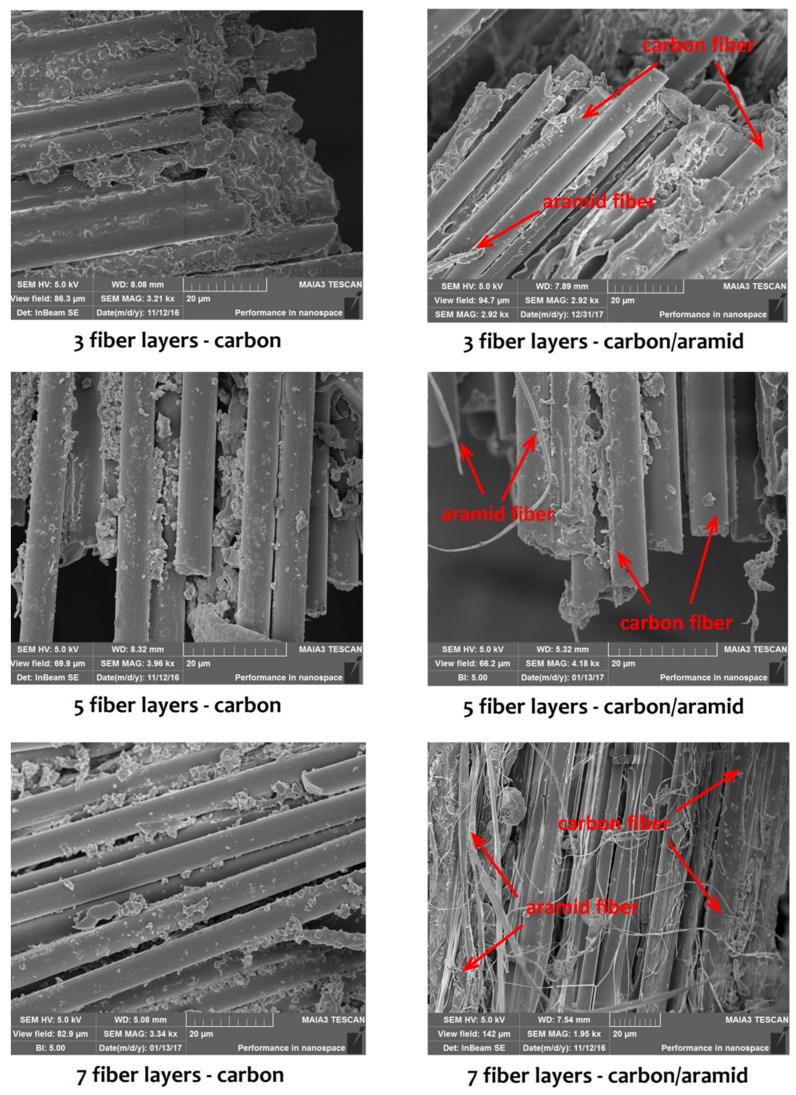
Scanning electronic microscope (SEM) micrographs of different patch fracture surfaces.

The scanning electronic microscope (SEM) micrographs of tensile fracture surfaces of traditional carbon fiber and carbon-aramid fiber sandwich composites are presented in Figure 5. From the images, it can be seen that there are obvious fiber full-outs on the fracture edges of both carbon-fiber-reinforced and carbon/aramid fiber-reinforced composite patches, while the phenomenon of less epoxy resin matrix being left among the fibers can be observed in the images of carbon-aramid fiber sandwich composites. As with the incorporation of aramid fiber layers, the content of carbon fibers gradually decreases from 2/3 to 4/7 when the lay numbers increase from 3 to 7. The tensile strength and modulus of aramid fiber is lower than carbon fiber, so the interface bonding between the epoxy resin matrix and aramid fiber is lower than that of carbon fiber, to some extent. Consequently, the tensile fracture of sandwich composite tends to occur after the increase of aramid fiber content. In addition, the interlaminar shear strength of carbon-aramid fiber sandwich composites is lower than that of carbon fiber composites, and as a result, the interface bonding of sandwich composites is also weakened with the increase of aramid fiber content. Further, for the carbon/aramid sandwich composite patches, two types of fibers with totally different sizes and discriminating properties could be recognized easily and clearly in the figures. Both the fiber pull-out and fiber separations show that the strengthening effect of aramid fiber layers on bending performance of composites is accompanied by the negative impact on surface conditions and bonding efficiencies among fibers and the matrix. Thus, maintaining the qualified tensile properties after the incorporation of the aramid fiber layers is vital in the structure optimization of bonded composite repair technology in this study.

In summary, based on the results of mechanical performance, including bending and tensile properties of the laminar composite patches above, it can be concluded that the sandwich structure, which inserts aramid fiber layers into the interlayers of carbon-fiber-reinforced laminar composite, improved the bending and further bending performance of the composite patch. From the bending tests of the composites, it is obvious that the bending strength as well as the modulus of structure-optimized laminar patches are strengthened effectively. However, the tensile behaviors are weakened to some extent. It can also be found that the composite patches, no matter if the composites are reinforced by traditional carbon fibers or sandwich structure carbon-aramid fibers, achieve the best mechanical behaviors when their fiber layer numbers amount to three in this study. Hence, to evaluate and compare the different repair performances between traditional and sandwich composite patches, both of the two types of composite patches with three fiber layers were chosen as the ultimate repair patches to continue further bonded repair experiments.

### 3.3. Majorization Effect of Sandwich Composite Patches on Repair Efficiency Improvement of Bonded Structures

It is known that the mechanical behaviors of bonded composite patches greatly affect the repair efficiency. Composite patches with higher tensile strength can bear the external forces loaded on the cracked plate more effectively, making the repaired structure more durable under external loading. However, there are certain limitations for the composite patches in the mechanical strengthening of cracked structures due to the constant tensile strength values of aluminum alloy plates. This means that the improving effect of high-strength composites would be limited once the tensile strength value of the bonded patch becomes larger than that of the aluminum alloy plate, making the alloyed plate the vulnerable component to failure. Thus, for the bonded composite patches, of which the mechanical strength is definitely stronger than that of the aluminum plate, there is little difference in the repair efficiency for composites being used as repair patches. In our study, according to the experimental results, the tensile strength of the aluminum alloy plate is around 358.9 MPa, while the composites reinforced by carbon and carbon-aramid fibers are 1.04 GPa and 0.86 GPa, respectively. This implies that even though the incorporation of aramid fiber would weaken the tensile performance of the composite to a certain extent, the tensile strength of the carbon-aramid fiber sandwich composite patch still maintains a high level compared to the aluminum alloy plate. This kind of high tensile strength promotes the feasibility and effect of the structural optimization design of composite patches. 

**Figure 6:**
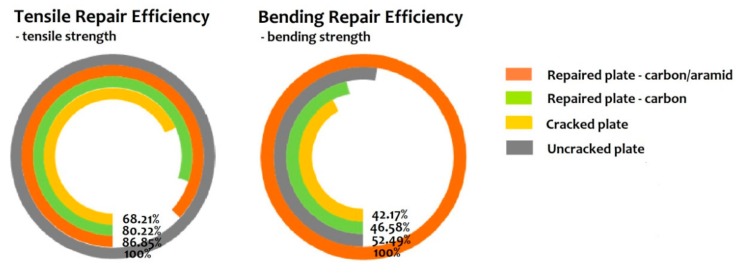
Pie charts of tensile and bending strength repair efficiency of the cracked plate repaired by the 3-layer sandwich composite.

Table 1 lists the bending and tensile strength of the aluminum alloy plate, cracked aluminum alloy plate, repair structure bonded by carbon-fiber-reinforced composites, and repair structure bonded by the carbon-aramid fiber sandwich composite. It can be seen from the table that the bending and tensile performance of the cracked aluminum plate are effectively recovered after the repair with bonded composite patches. The repair structure bonded by the carbon-aramid fiber sandwich composite exhibits excellent bending and tensile strength values of 1153.1 MPa and 311.7 MPa, respectively, much higher than the unrepaired aluminum alloy plate at 486.3 MPa and 244.8 MPa. Notably, the bending strength of the sandwich composite repair structure is even higher than that of the uncracked aluminum alloy plate. For further analysis, the recovery percentage pie charts for the repaired structures are presented in Figure 6. It can be seen that for tensile strength, the recovery percentages of the repair structures bonded by traditional carbon fiber composite patches and the carbon-aramid fiber sandwich composite patches occupy 80.22% and 86.85% percent of the uncracked aluminum alloy plate, respectively. On the other hand, it is interesting that the bending strength of the cracked aluminum alloy plate is dramatically increased after the bonding of the sandwich composite patch, far surpassing the uncracked plate and recovering 190% of its strength.

On the basis of the results mentioned in Table 1 and Figure 6 above, it is easy to see that for the cracked aluminum plate, the bending and tensile performance were recovered and even highly strengthened by bonding with the sandwich composite patches, which are reinforced by carbon-aramid fiber layers. In comparison with the carbon-fiber-reinforced composite patch, the optimized sandwich composite displays a considerable and higher repair efficiency for the recovery of static mechanical performance for the central cracked aluminum plate, especially in its bending properties. Such outstanding repair performance for the sandwich composite can be attributed to the shear strengthening effect of the incorporation of flexible aramid fiber layers inside the composite patches. The shear deformation capability of the laminar composite can be greatly improved under the premise that the tensile properties of the patches are sufficiently high. The incorporation of aramid fiber layers can help the composite patches to adhere to the cracked aluminum alloy plate more easily. Furthermore, it also makes the composite patches more flexible in adapting to the deformation of the repair plate under external shear forces. Therefore, the cracked aluminum alloy plate bonded by the carbon-aramid fiber sandwich composite patch displays excellent performance under the bending load, showing its obvious optimization for repair efficiency. According to the related experimental results, it is worth mentioning that even though the tensile performances of the carbon-aramid fiber sandwich composite patches are lower than that of single carbon-fiber-reinforced composite patches, the repaired structure bonded by the sandwich patch shows better anti-tensile behaviors. This can be reasonably explained by the fact that under tensile load, the central cracked aluminum plate would be partly deformed due to its structural deficiency. This would finally lead to certain spatial warping and torsion phenomenon around the cracked area, introducing the vectors out of the tensile plane and causing the bending deformations of the plate to a certain extent. Thus, the bonded sandwich patch, with good flexibility, can better adhere to the cracked aluminum alloy plate under complex loading conditions. When bearing the external forces, including both tensile and bending loads, the sandwich patch presents a more effective and stable repair solution compared to the single carbon-fiber-reinforced composite patch.

## 4. Conclusions

The microwave cured carbon-aramid fiber sandwich composite was designed and prepared for the bonded patches to repair a central cracked aluminum alloy plate. The results of bending and tensile experiments showed that the sandwich composite patch structure presents higher bending strength and modulus but lower tensile strength and modulus than those of the patches with single carbon fiber fabric layers. For the sandwich composite patches, the incorporation of aramid fiber layers can effectively improve the bending behaviors of laminar composites due to the flexibility and small size of the aramid fibers. Both traditional single carbon fiber and carbon-aramid fiber sandwich composite patches with three fiber layers exhibited the best mechanical properties. Meanwhile, the tensile and bending performance of the composite patches decreased with the further increase of fiber layer numbers. For the repaired structures, the static mechanical performance of the cracked aluminum alloy plate was strengthened and recovered by the bonding with repair patches. The recovery percentages for tensile and bending strength of the cracked plate bonded with optimal three-fiber-layer carbon-aramid fiber sandwich patches reached over 86% and 190%, respectively, compared to the uncracked ones, presenting outstanding repair effects in comparison with the traditional single carbon fiber patches.

## Figures and Tables

**Table 1:** Tensile and bending performance of the aluminum alloy plate, cracked aluminum alloy plate, and repaired structure.

Sample	Bending Strength (MPa)	Tensile Strength (MPa)
Aluminum alloy plate	605.3 (±13.1)	358.9 (±11.9)
Cracked aluminum alloy plate	486.3 (±9.4)	244.8 (±7.2)
Repaired plate-carbon fiber	537.1 (±34.2)	287.9 (±13.7)
Repaired plate-carbon/aramid fiber	1153.1 (±29.8)	311.7 (±22.6)
